# Preclinical Evaluation of the Oncolytic Vaccinia Virus TG6002 by Translational Research on Canine Breast Cancer

**DOI:** 10.1016/j.omto.2020.08.020

**Published:** 2020-09-02

**Authors:** Jérémy Béguin, Johann Foloppe, Christelle Maurey, Eve Laloy, Julie Hortelano, Virginie Nourtier, Christelle Pichon, Sandrine Cochin, Pascale Cordier, Hélène Huet, Eric Quemeneur, Bernard Klonjkowski, Philippe Erbs

**Affiliations:** 1 UMR Virologie, INRA, Ecole Nationale Vétérinaire d’Alfort, ANSES, Université Paris-Est, Maisons-Alfort 94700, France; 2 Transgene S.A., 400 Boulevard Gonthier d’Andernach, Parc d’innovation, CS80166, Illkirch-Graffenstaden Cedex 67405, France; 3 Service de Médecine Interne, Ecole Nationale Vétérinaire d’Alfort, Université Paris-Est, Maisons-Alfort, 94700, France; 4 Laboratoire d’Anatomo-cytopathologie, Biopôle Alfort, Ecole Nationale Vétérinaire d’Alfort, Université Paris-Est, Maisons-Alfort 94700, France

**Keywords:** oncolytic virotherapy, vaccinia virus, TG6002, *FCU1*, suicide gene therapy, mammary tumor, dog, translational research, 5-fluorouracil

## Abstract

Oncolytic virotherapy is a promising therapeutic approach for the treatment of cancer. TG6002 is a recombinant oncolytic vaccinia virus deleted in the thymidine kinase and ribonucleotide reductase genes and armed with the suicide gene *FCU1*, which encodes a bifunctional chimeric protein that efficiently catalyzes the direct conversion of the nontoxic 5-fluorocytosine into the toxic metabolite 5-fluorouracil. In translational research, canine tumors and especially mammary cancers are relevant surrogates for human cancers and can be used as preclinical models. Here, we report that TG6002 is able to replicate in canine tumor cell lines and is oncolytic in such cells cultured in 2D or 3D as well as canine mammary tumor explants. Furthermore, intratumoral injections of TG6002 lead to inhibition of the proliferation of canine tumor cells grafted into mice. 5-fluorocytosine treatment of mice significantly improves the anti-tumoral activity of TG6002 infection, a finding that can be correlated with its conversion into 5-fluorouracil within infected fresh canine tumor biopsies. In conclusion, our study suggests that TG6002 associated with 5-fluorocytosine is a promising therapy for human and canine cancers.

## Graphical Abstract

**Figure:**
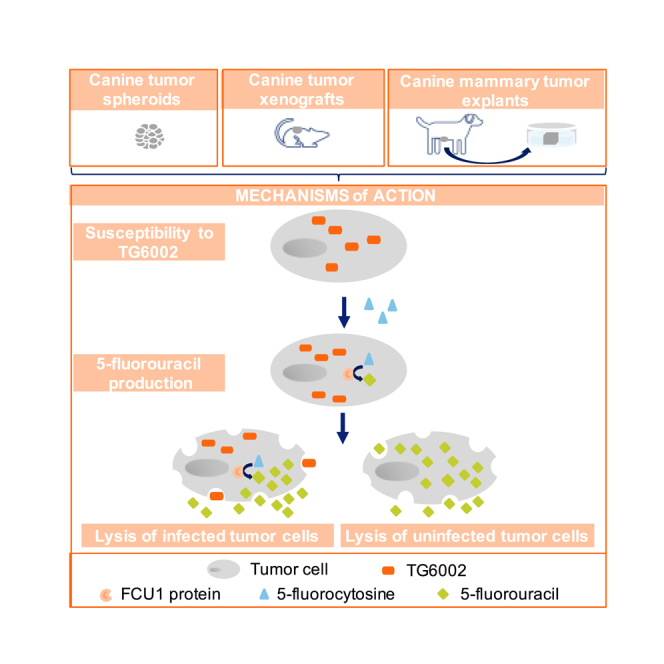

## Teaser

TG6002 is a double-deleted vaccinia virus expressing FCU1 that converts 5-fluorocytosine into 5-fluorouracil. This study demonstrates oncolytic properties of TG6602 in 2D and 3D cultured canine mammary cell lines, in a xenograft model, and in canine mammary tumor explants. Moreover, it establishes 5-fluorouracil production in fresh canine tumor biopsies.

## Introduction

Cancer is one of the leading causes of death for both humans and pet animals. Current treatment strategies involve surgery, radiation, chemotherapy, and immunotherapy. Existing therapies often fail to cure cancer; therefore, the development of novel ones is needed. Oncolytic virotherapy is one of the promising approaches. Indeed, a genetically engineered herpes simplex virus (Imlygic) has been approved by the European Medicines Agency and the U.S. Food and Drug Administration for the local treatment of unresectable melanoma.^1^ Oncolytic viruses are naturally occurring or engineered viruses that selectively infect and replicate in cancer cells, inducing oncolysis and antitumoral immune responses while sparing healthy tissue. Targeting of these viruses to cancer cells is based on natural tropism and the deletion of viral genes required for replication in non-dividing cells. A wide array of viruses has been studied in preclinical and clinical studies, including adenovirus, vaccinia virus (VACV), herpes virus, vesicular stomatitis virus, and measles.^2^^,^^3^ VACV belongs to the *Poxviridae* family, which comprises large, double-stranded DNA viruses that replicate in the cell cytoplasm. Its ability to multiply, lyse host cells, evade immune responses, and spread across a broad range of mammalian hosts makes it a valid candidate for oncolytic virotherapy.^4^ Moreover, an intrinsic tumor tropism has been reported for VACV.^5^ In addition, VACV has a large genome that can host transgenes to increase its oncolytic potency.^4^ VACV also displays biosafety due to the absence of DNA integration and the availability of effective antiviral agents.^6^ To be used as an oncolytic agent, genomic modifications are necessary to selectively target tumor cells and restrict virus propagation to the tumor. VACV can be easily engineered, and numerous viral genes have been deleted to generate a safe and efficient oncolytic vector.^7^ One of the outstanding candidates developed so far is Pexa-Vec (JX-594) a thymidine-kinase (*TK*)-deleted Wyeth strain of VACV expressing the granulocyte-macrophage colony-stimulating factor (GM-CSF) cytokine to activate immune cells at the tumor site.^8^^,^^9^ Another highly attenuated oncolytic VACV, designated TG6002, has been developed.^10^ TG6002 is a Copenhagen strain VACV deleted in two genes: *TK* (*J2R*) and a subunit of the ribonucleotide reductase (*RR*; *I4L*). TG6002 has demonstrated strong tumor selectivity and retained a full capacity to replicate and lyse human cancer cell lines.^10^ *TK*/*RR*-deleted VACV replication depends on the cellular pool of TK and RR and, thus, on the expression of cellular TK and RR, which is known to be overexpressed in tumor cells. The double deletion leads to an improved safety profile as a result of very high attenuation in healthy tissues.^10^ TG6002 is armed with the suicide gene *FCU1*, which encodes a bifunctional chimeric protein that catalyzes the conversion of nontoxic 5-fluorocytosine (5-FC) into toxic metabolites 5-fluorouracil (5-FU) and 5-fluorouridine monophosphate.^11^ The expression of the *FCU1* gene by the virus allows targeted chemotherapy within the tumor.^10^

Much of the research on human diseases relies on animal models. Due to several advantages, the mouse is the most frequently used model. Indeed, technologies are available that allow the modulation of the expression of mouse genes in the entire organism or in selected tissues.^12^ A major limitation of the mouse model is that tumors are grafted or induced and do not arise spontaneously as in humans or dogs. Furthermore, unlike for human cancers for which disease is polygenic, cancers induced in mouse models involve one or only a few genes. For these reasons, mouse models are not excellent models of human cancers. Spontaneous canine cancers, such as mammary cancers, appear to be more relevant models of human cancers. Canine mammary tumors are naturally occurring cancers that have several features in common with human breast cancer.^13^ Indeed, clinical presentation, hormonal etiology, environment, histological features, molecular profiles (steroid hormone receptors, proliferation markers, epidermal growth factor, p53 suppressor gene mutations, cyclooxygenases or metalloproteinases), and response and resistance to therapy are quite similar.^14^, ^15^, ^16^, ^17^, ^18^, ^19^ Canine mammary tumors account for almost 50% of all canine neoplasms. Thus, the increased prevalence facilitates timely completion of clinical studies.^20^ Thereby, the evaluation of the potency of oncolytic viruses on spontaneous canine cancers should be valuable for assessing their potential benefit in human medicine.

The first objective of this study was to examine the *in vitro* susceptibility and rate of replication of double-deleted Δ*I4L*Δ*J2R* VACV on 2D and 3D canine tumor models. The second objective was to evaluate the effects of TG6002 in a subcutaneous canine mammary carcinoma xenograft model. Finally, we assessed the susceptibility and oncolytic potency of TG6002 on canine tumor explants.

## Results

### Susceptibility of Human and Canine Tumor Cell Lines

**Figure 1:**
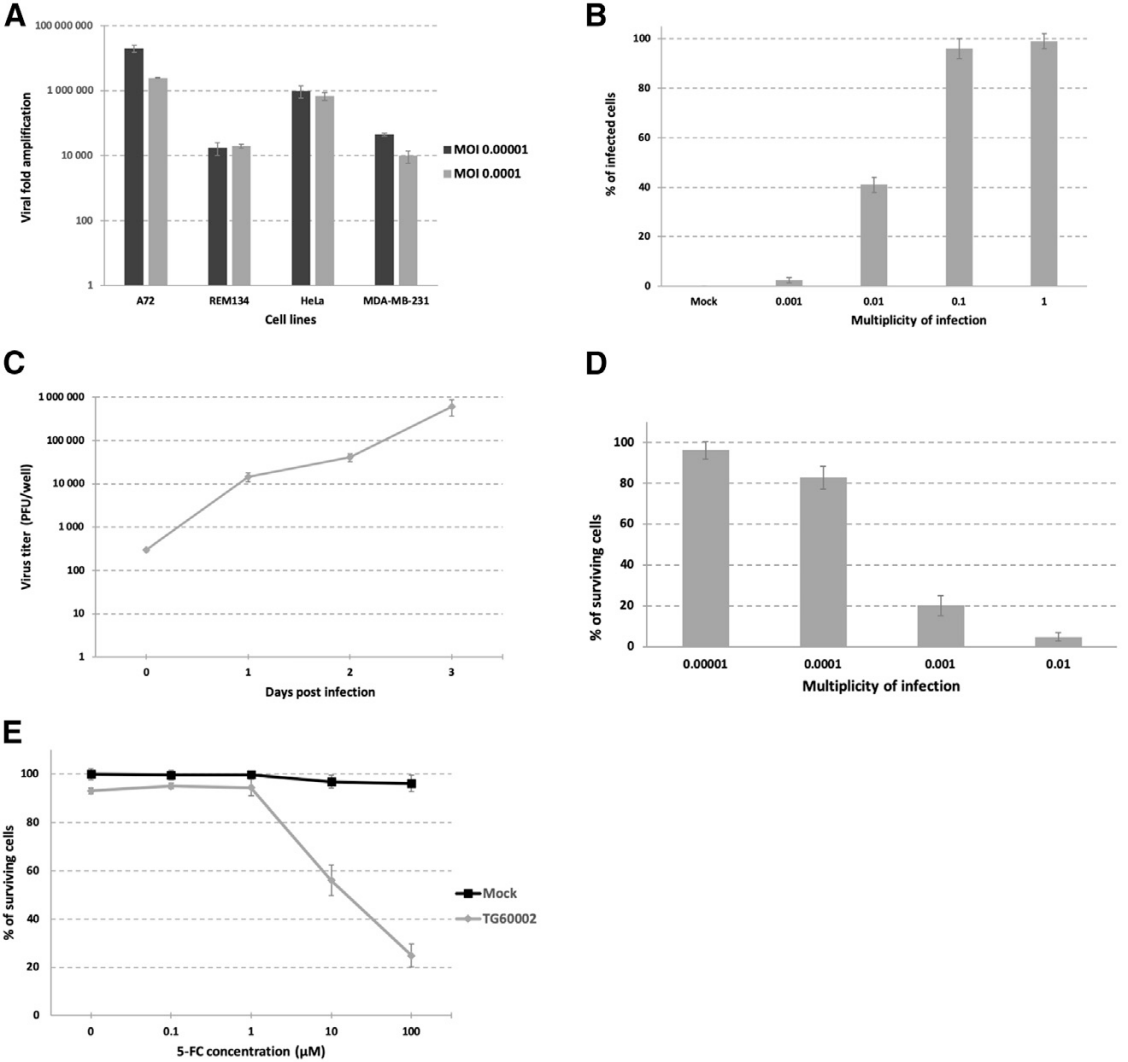
Characterization of *In Vitro* Infection, Replication, and Oncolytic Activity of the Double-Deleted VACV on Canine Cancer Cells Using 2D Monolayer Cultures (A) Amplification factor of VVTG17990 in two canine tumor cell lines (A72 and REM134) and two human tumor cell lines (HeLa and MDA-MB-231) infected at MOIs of 10^−5^ and 10^−4^ and collected 4 days post-infection. The results are presented as a mean of triplicate experiments ± SD. (B) Susceptibility of REM134 canine tumor cell line to VACV infection. REM134 tumor cells were infected at the indicated MOI with VVTG17990, and the percentage of GFP-positive cells was determined by cytometry at 24 h post-infection. The results were obtained from three separate experiments ± SD. (C) Viral replication kinetics of TG6002 on canine tumor cells. Replication capacity was analyzed through viral growth curve by recovering infected REM134 monolayers (300 PFUs per well) at different time points. The titers of progeny virus were determined by serial dilution titration on Vero cells. The results are presented as a mean of triplicate experiments ± SD. (D) Oncolytic effect of the double-deleted VACV on REM134 canine tumor cells. Cells were infected at MOIs of 10^−5^, 10^−4^, 10^−3^, or 10^−2^ with TG6002, and cell viability was measured 5 days later by trypan blue exclusion. The results are presented as a mean of triplicate experiments ± SD. (E) Combination of oncolytic and prodrug cytotoxicity. REM134 canine tumor cells were infected at a MOI of 10^−4^ with TG6002. After 48 h, 5-FC was added in a range of concentrations, and cell survival was determined 3 days later by trypan blue exclusion. Results were standardized against values for wells lacking virus and drug, which represented 100% viability. Values are represented as means ± SD of three individual determinations.

To evaluate the relative ability of double-deleted VACV to enter and produce infectious progeny in canine tumor cells, we performed a replication assay in a canine mammary carcinoma cell line (REM134) and a canine tumor cell line (A72) in parallel with two well-known human cancer cell lines, HeLa and MDA-MB-231.^21^^,^^22^ As shown in Figure 1A, the A72 cell line produced the highest virus yield with an amplification factor of 2 × 10^7^ after infection at a multiplicity of infection (MOI) of 10^−5^; 20 times more than the amplification factor in HeLa, 500 times more than in MDA-MB-231, and 1,000 times more than in REM134. The results were equivalent at a MOI of 10^−4^. The REM134 cell line was the least productive for VACV replication but with an amplification factor similar to that obtained in MDA-MB-231. The canine cell lines used in this assay were able to amplify double-deleted VACV as efficiently as human tumor cell lines. These results filled a first requirement in considering the value of recombinant oncolytic VACV in canine cancer. We decided to pursue studies with the less permissive cell line (REM134) to validate the ability of TG6002 to be used as a therapy in veterinary medicine.

### VACV Infects, Replicates, and Induces Lysis in REM134 Cells *In Vitro*

The overall permissiveness of REM134 cells for VACV was determined using a Δ*I4L*Δ*J2R*VACV expressing GFP (VVTG17990) and measuring the percentage of GFP-positive cells at early time points after infection. REM134 cells were susceptible to VACV infection and showed a high transduction efficiency (greater than 90%) at both MOIs of 10^−1^ and 1 (Figure 1B). To evaluate the kinetics of TG6002 replication in canine tumor cell lines, we measured the virus yields produced after 24, 48, and 72 h of infection in REM134 cells at a MOI of 10^−3^. A constant increase in yield was observed over the course of the experiment to reach, 72 h post-infection, an amplification factor between 4,000 and 5,000 (Figure 1C).

The REM134 cancer cell line was also used in a cell-killing assay to assess the anti-tumor potency of TG6002 at various MOIs. TG6002 was able to kill canine tumor cells in a dose-dependent manner (Figure 1D). Five days post-infection, with TG6002 used at MOIs of 10^−5^ and 10^−4^, cell lysis was low. More than 95% of tumor cells were killed at a MOI of 10^−2^, and more than 80% lethality was detected at a lower MOI of 10^−3^, indicating the ability of TG6002 to destroy canine tumor cells (Figure 1D).

### TG6002/5-FC Cell Killing by Combination of Prodrug Activation with Viral Oncolysis

Prior to the evaluation of the oncolytic effect of TG6002 in the presence of the prodrug 5-FC, the sensitivity of REM134 to 5-FU was examined at different concentrations to ensure the ability of the chemotherapeutic agent to induce toxicity in the tumor canine cell line. Concentrations of 1 μM and 10 μM 5-FU led to a 90% reduction of cell viability, and a half-maximum inhibitory concentration (IC_50_) of the order of 0.5 μM was determined, demonstrating 5-FU efficacy on this canine tumor cell line (data not shown). We next compared the anti-proliferative activity of TG6002 alone or combined with 5-FC treatment (Figure 1E). REM134 cells were infected with a suboptimal dose of TG6002 (MOI of 10^−4^), inducing only 10% of cytotoxicity. 5-FC, added in the culture medium 48 h post-infection, largely killed REM134 cells infected by TG6002 in a prodrug dose-dependent manner. In the presence of 10 μM and 100 μM 5-FC, cell viability was reduced by 40% and 80%, respectively, compared to uninfected cells treated with the same amount of 5-FC (Figure 1E). These results indicate that TG6002 with 5-FC acquired an enhanced *in vitro* anti-tumor activity in the canine tumor cell line REM134, thanks to the conversion of the 5-FC to the cytotoxic agent 5-FU.

### Evaluation of TG6002 in Canine Tumor Spheroids

**Figure 2:**
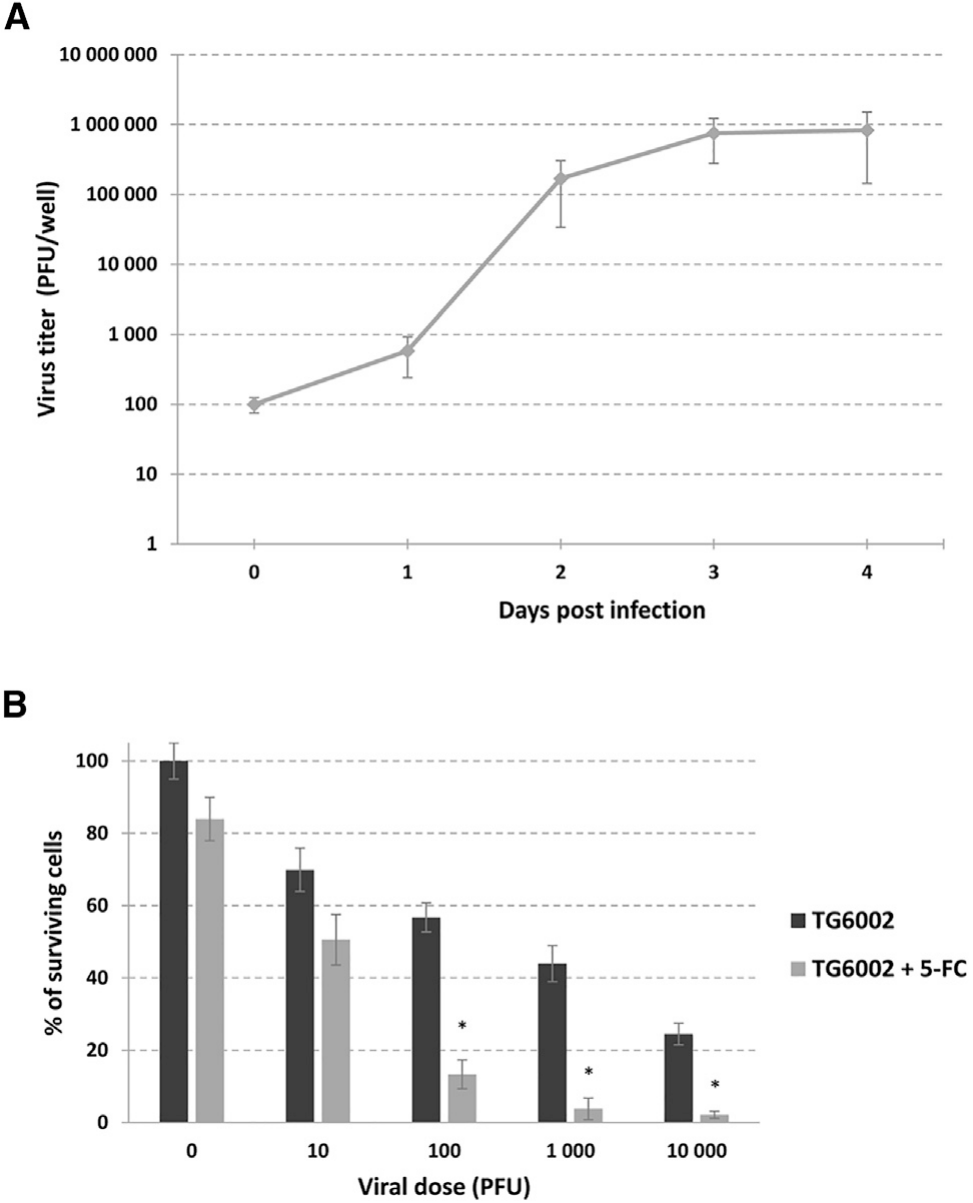
Characterization of Replication and Oncolytic Activity of TG6002 on a 3D *In Vitro* REM134 Model (A) Viral replication kinetics of TG6002 on REM134 spheroids. Replication capacity was analyzed through viral growth curve by recovering infected REM134 spheroids (100 PFUs per well) at different time points. The titers of progeny virus were determined by serial dilution titration on Vero cells. The results are presented as a mean of triplicate experiments ± SD. (B) Combination of oncolytic and prodrug cytotoxicity on REM134 spheroids. Spheroids were infected with the indicated doses of TG6002. After 48 h, 5-FC was added at 1 mM, and cell survival was determined 8 days later using the CellTiter-Blue Cell Viability Assay. Results were standardized against values for wells lacking virus and drug, which represented 100% viability. The asterisks indicate a significant difference (p < 0.05) between groups (Student’s t test). Values are represented as means ± SD of three individual determinations.

We investigated the therapeutic potential of TG6002 in a 3D culture system. First, we generated spheroids from the canine mammary carcinoma cell line REM134 by using ultra-low attachment plates. After confirmation of spheroid formation by microscopic visualization at day 4, TG6002 was added into the spheroid cultures, and the kinetics of viral multiplication were determined over a 4-day period (Figure 2A). The data showed that a significant amount of virus was obtained with an amplification factor of around 10,000 in 3 days (Figure 2A). In addition, we determined cell viability in the 3D culture system using the CellTiter-Blue Cell Viability Assay and found that TG6002 induced cytotoxicity in REM134 spheroids in a dose-dependent manner (Figure 2B). Moreover, the results indicated that spheroids treated with 10^2^ PFUs, 10^3^ PFUs, and 10^4^ PFUs of TG6002 and 5-FC showed a significant decrease of viability compared with the spheroids treated only with TG6002 (p < 0.05) (Figure 2B).

### Immunohistochemical Analyses of Canine Tumor Xenografts

**Figure 3:**
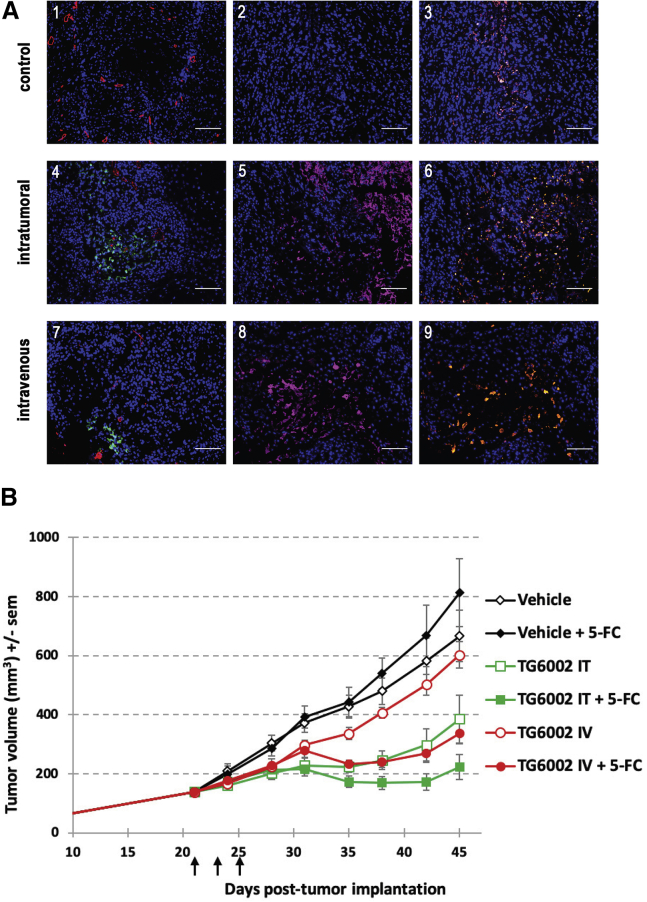
*In Vivo* Anti-tumor Activity of VACV on a REM134 Canine Tumor Xenograft Model (A) *In vivo* detection of VACV and apoptosis in the REM134 xenograft tumors. Untreated mice (1, 2, and 3) and mice treated intratumorally (10^6^ PFUs; 4, 5, and 6) or intravenously (10^7^ PFUs; 7, 8, and 9) with VVTG17990 were sacrificed at day 14 post-infection, and macroscopic tumors were removed, formalin fixed, and analyzed by immunohistochemistry. Cellular DNA was stained in blue with DAPI (1–9), GFP was stained in green (1, 4, and 7), CD31 was stained in red (1, 4, and 7), VACV was stained in purple (2, 5, and 8), and activated caspase-3 was stained in orange (3, 6, and 9). Scale bars, 100 μm. (B) Mean tumor volume after intratumoral or systemic administration of TG6002 with 5-FC. Mice bearing REM134 subcutaneous xenografts were treated with three intratumoral (10^6^ PFUs) or intravenous (10^7^ PFUs) injections of TG6002 (indicated by vertical arrows). Three days after the last virus injection, the animals were treated twice daily with *per os* administration of water or 5-FC (200 mg/kg/day) for 3 weeks. The data represent mean ± SD of 11 animals.

In mice, subcutaneous REM134 tumors are organized into glandular structures with islets of tumor cells surrounded by connective tissue (data not shown). CD31 staining confirmed the vascularization of the xenograft (Figures 3A1, 3A4, and 3A7). Expression of GFP was observed in the REM134 tumors 2 weeks after intratumoral (10^6^ PFUs) or intravenous (10^7^ PFUs) injection of VVTG17990 (Figures 3A4 and 3A7, respectively). For both intratumoral and intravenous protocols with VVTG17990, immunohistochemical analyses using cleaved caspase-3 immunostaining confirmed areas of apoptosis in tumor tissues (Figures 3A6 and 3A9, respectively). Furthermore, VACV immunostaining (Figures 3A5 and 3A8) was observed in the same location as caspase immunostaining, confirming that lysis and apoptosis were induced by VACV. For both intratumoral and intravenous injections, VACV was detected in numerous sites within the tumor, showing that VACV could replicate and invade the tumor (Figures 3A5 and 3A8, respectively).

### Antitumor Effect of TG6002/5-FC in a Canine Xenograft Tumor Model

Nude mice bearing established REM134 xenografts were treated with intratumoral (10^6^ plaque-forming units [PFUs]) or intravenous (10^7^ PFUs) injections of TG6002. The animals were then treated with *per os* administration of water or 5-FC for 3 weeks. As shown in Figure 3B, the administration of 5-FC alone had no effect on REM134 tumor growth. Intravenous injections of TG6002 resulted in a slight inhibition of tumor growth compared with the control groups (no treatment or 5-FC alone). In contrast, the 5-FC treatment significantly improved TG6002 antitumor activity when compared to TG6002 alone (p < 0.05), indicating that the FCU1/5-FC approach compensated for the lack of potent oncolytic activity of TG6002 injected intravenously in this model (Figure 3B). In this canine xenograft tumor model, TG6002 injected intratumorally, without 5-FC treatment, induced a significant antitumor effect, with a reduction of 50% of tumor volume, as compared to the control groups (p < 0.05) (Figure 3B). The administration of 5-FC enhanced the antitumor activity of TG6002 injected intratumorally, resulting in a strong inhibition of tumor growth as compared to the control groups (p < 0.05), with a tumor volume reduction greater than 70%. For mice treated intratumorally with TG6002, from day 35 after tumor implantation, there was a significant difference in tumor size between the groups with and without 5-FC administration (p < 0.05). Thus, our *in vivo* results suggest that TG6002 with 5-FC can effectively inhibit the growth of canine tumor in a subcutaneous xenograft mice model.

### Evaluation of Canine Neoplastic Explants

Three canine grade I^23^ complex tubular mammary adenocarcinomas with a Ki67 of 3.48%, 6.26%, or 7.02% were used in this study to assess susceptibility to VACV and oncolytic potency of TG6002 with 5-FC. Tumor samples were obtained from dogs undergoing therapeutic surgical excision, at the National Veterinary School of Alfort, with pet owner consent. Fresh biopsy samples of canine mammary tumors were collected immediately after surgery.

**Figure 4:**
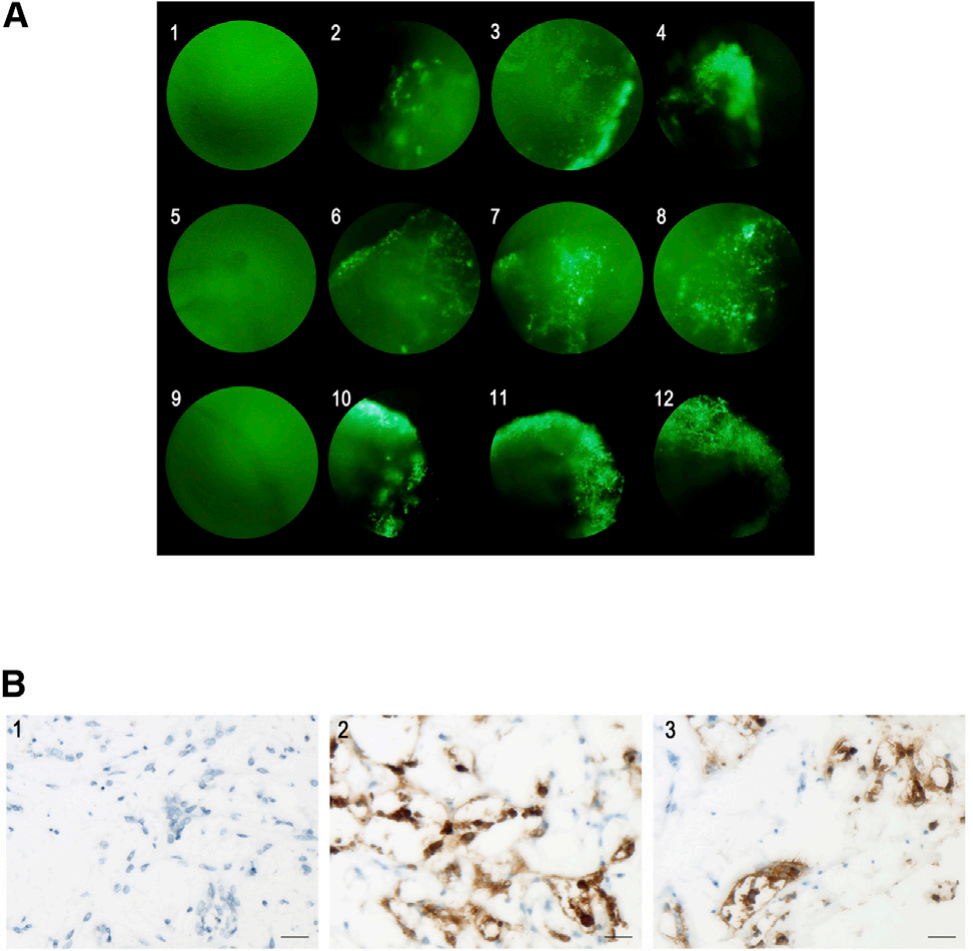
*In Vitro* Evaluation of VACV Transduction in Canine Mammary Tumor Explants (A) Transduction of VACV in canine mammary tumor explants by fluorescence microscope detecting GFP. Three biopsies were infected with 10^6^ PFUs of VVTG17990 per well, and transduction was monitored 2, 3, and 4 days after infection. From left to right, each column corresponds to days 0, 2, 3, and 4. From top to bottom, each line fits to a canine mammary tumor explant (first tumor, 1–4; second tumor, 5–8; and third tumor, 9–12). GFP expression was observed and increased during 4 days after infection with VVTG17990, confirming the susceptibility of three canine grade I tubular complex mammary adenocarcinomas to VACV (magnification, 200×). (B) Microphotographs: immunohistochemistry for VACV on uninfected canine mammary tumor explant (1) and two canine mammary tumor explants infected with 10^6^ PFUs of VVTG17990 (2 and 3); viral antigen is detected in the cytoplasm of tumoral cells (brown staining). Scale bars, 20 μm.

To assess the susceptibility of canine mammary tumors to VACV, one sample of each tumor was infected with VVTG17990. GFP fluorescence was observed at the periphery of infected explants after 48 h (Figures 4A2, 4A6, and 4A10), and infection progressed toward the center until the fourth day for all samples (Figures 4A4, 4A8, and 4A12). Immunohistochemical analyses confirmed the infection by VVTG17990 (Figure 4B).

**Figure 5:**
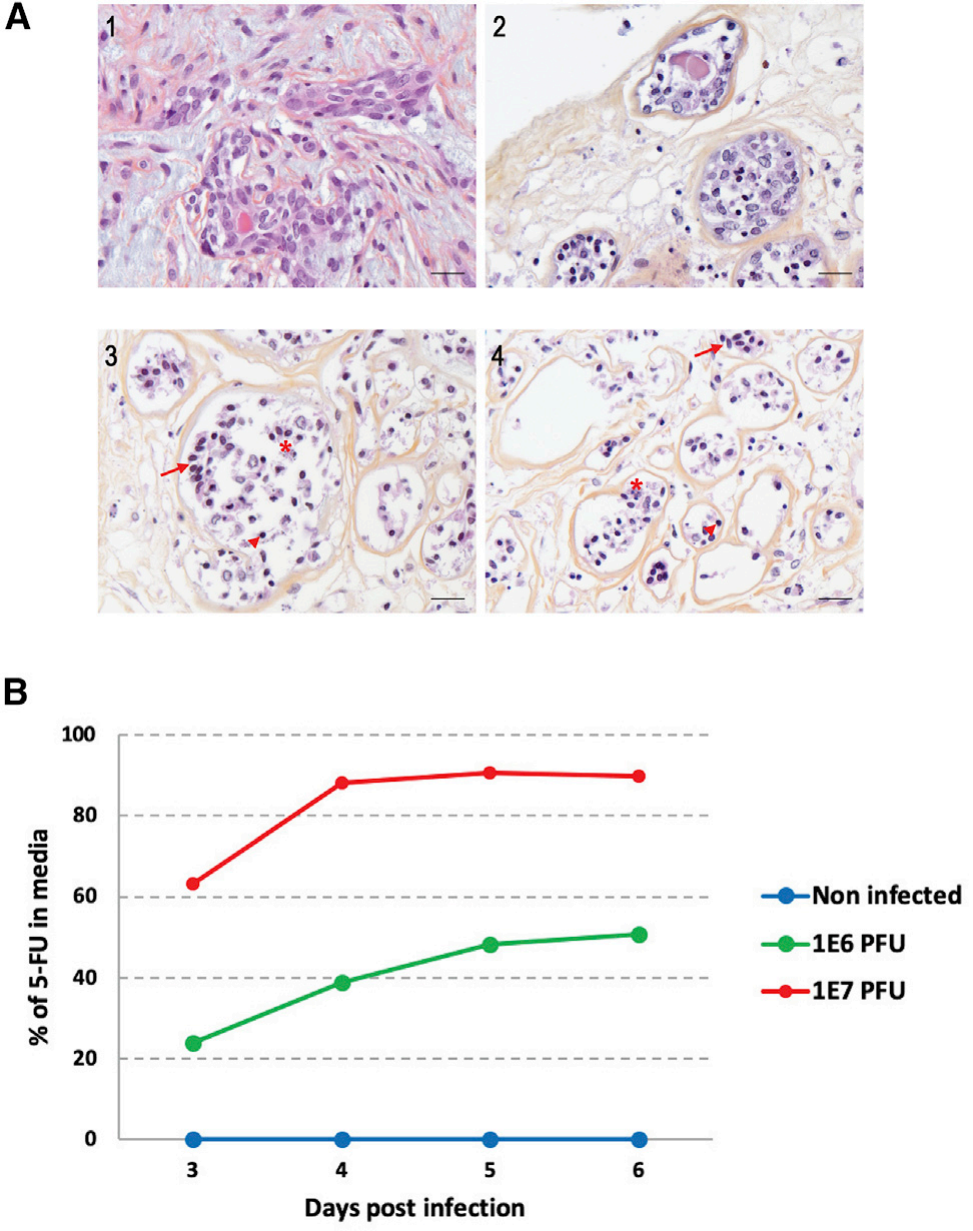
*In Vitro* Evaluation of the Oncolytic Potency of TG6002 with 5-FC in Canine Mammary Tumor Explants (A) Histological microphotographs of uninfected canine mammary tumor explants and infected canine mammary tumor explants with TG6002 and 5-FC. (1) Microphotograph: biopsy of canine mammary tumor collected after surgical excision diagnosis of tubular complex mammary adenocarcinoma, grade I, without necrosis. (2) Microphotograph: explant of canine mammary tumor cultured in culture medium for 6 days after surgical collection. (3 and 4) Microphotographs: explants of canine mammary tumor cultured with TG6002 and 5-FC. Six days after infection by TG6002—10^6^ PFUs (3) or 10^7^ PFUs (4)—with 5-FC, nuclear pyknosis (arrowheads), cell-to-cell detachment (arrows), and cell debris (asterisks) in the lumen of tubules were noticed. Tumor necrosis ranged from 75% to 100%, regardless of TG6002 dosage. Hematoxylin-eosin-saffron (HES) staining was used. Scale bars, 20 μm. (B) 5-FU generated by canine mammary tumor explant infected by TG6002 upon conversion of 5-FC. A canine mammary tumor biopsy was infected with 10^6^ or 10^7^ PFUs of TG6002 and then incubated with 1 mM 5-FC from day 2 to day 6 post-infection. The relative concentration of 5-FC and 5-FU in the culture supernatant was measured by high-performance liquid chromatography. The results are expressed as the percentage of 5-FU in the media relative to the total amount of 5-FC plus 5-FU.

The level of necrosis was measured on three uninfected samples to evaluate the effect of culture conditions. The percentage of spontaneous necrosis was between 25% and 50% for one biopsy and between 50% and 75% for two biopsies (Figure 5A2). A percentage of necrosis between 25% and 75% was estimated for three canine mammary explants with 5-FU (data not shown).

One sample of 6.26% and one sample of 7.02% Ki67 grade I complex tubular mammary adenocarcinoma were infected, respectively, with 10^6^ PFUs or 10^7^ PFUs of TG6002 for 6 days. After infection with TG6002 and 5-FC, necrosis was more extensive as compared to the control sample (Figures 5A3 and 5A4). Necrosis was estimated to represent 75%–100% of biopsies, regardless of the dosage of TG6002 associated with administration of 5-FC (Figures 5A3 and 5A4).

To evaluate FCU1 activity in the infected biopsies, and knowing that 5-FC and 5-FU are diffusible across cells membranes,^11^ we measured the concentration of 5-FC and 5-FU in the culture supernatants. Analysis of the cell culture supernatants by high-performance liquid chromatography revealed a decrease of 5-FC and an increase in the amount of 5-FU in the extracellular medium of canine mammary tumor explants infected by TG6002 with 5-FC (Figure 5B). This result indicates that TG6002 infects tumor biopsies and produces large amounts of FCU1 that are able to catalyze 5-FC into 5-FU. As expected, the production of 5-FU in the supernatants was dependent on the dose of virus with, 5 days post-infection, 50% and 90% of 5-FC deaminated into 5-FU at 10^6^ PFUs and 10^7^ PFUs, respectively (Figure 5B). In contrast, no 5-FU was detected in the extracellular medium of non-infected biopsies (Figure 5B). Taken together, our data indicate that TG6002 can infect canine mammary tumor explants inducing expression of functional FCU1 protein.

## Discussion

Breast cancer is a major concern in human oncology. It represents the most prevalent cancer and the leading cause of cancer death in women worldwide.^24^ Indeed, in 2012, approximately 2 million women were diagnosed with breast cancer.^25^ As a result, the development of new treatments for breast cancer are needed. As in human oncology, canine mammary cancer remains the most common canine cancer, with an estimated annual incidence of 182 per 100,000 female dogs.^26^ There is some evidence to suggest that neutering bitches before the age of 2.5 years is associated with a significant reduction in the risk of malignant mammary tumors.^27^ Moreover, this risk may be reduced further by neutering before the first estrous cycle.^27^ A study established that bitches spayed before any estrous cycles had approximately 0.5% of the mammary cancer risk, those that had only one estrous cycle had 8%, and dogs that had two or more estrous cycles before neutering had 26%.^28^ Comparative oncological studies have successfully used canine mammary tumors as a suitable model for human breast cancer research.^29^, ^30^, ^31^, ^32^, ^33^, ^34^ Human and dog cancers share clinical and etiological similarities such as the age of onset, the spontaneous nature of the tumor, the hormonal etiology, and the course of the disease.^15^^,^^35^ Furthermore, many studies have reported significant similarities regarding the expression of molecular markers such as the overexpression of steroid hormone receptors, proliferation markers, epidermal growth factor, p53 suppressor gene mutations, cyclooxygenases, or metalloproteinases.^14^ In addition, similar histological patterns between the two species have been described for mammary cancers.^16^, ^17^, ^18^, ^19^ To improve the relevance of our experimental models, a canine mammary carcinoma cell line and fresh canine mammary tumor biopsies from dogs with spontaneous mammary carcinoma were used.

Previous studies have reported a significant inhibition of tumor growth in xenograft models after administration of oncolytic VACV expressing FCU1 with oral administration of 5-FC.^10^^,^^36^^,^^37^ In our study, a significant reduction of tumor growth was observed after intratumoral treatment with TG6002 associated with oral 5-FC administration. This emphasizes the relevance of the suicide gene *FCU1* to improve treatment response. Even though TG6002 is able to target tumors after intravenous administration, better results were noticed after intratumoral administration. Thus, this route of administration may be a better option in cancer treatment. Prior studies have demonstrated that the double-deleted VACV may also reduce tumor load at sites distant from its local administration,^38^ a finding that remains to be investigated in the case of canine tumors.

The use of cancer cell lines as experimental models is a common procedure in cancer research. Cell lines are generally cultured in 2D conditions. However, discrepancies with real-life situations may occur, particularly because of variability in cell morphologies, the inability to communicate with other cells, and failure to facilitate metabolic functions. A growing number of publications report the advantages of 3D culture compared to 2D culture. Cell cultures maintained in 3D conditions exhibit morphologic and proliferative features that are closer to the natural state.^39^ Indeed, spheroids can mimic the tumor microenvironment with high oxygen and nutrition supply at the rim and accumulation of metabolic end products in the core. This can better model neoplasia, with a proliferative periphery and quiescent or necrotic center. Moreover, cells cultured in 3D conditions exhibit cell interactions that can influence the oncolytic activity of the virus and the bystander effect induced by the suicide gene. The kind and extent of tumor cell interactions like gap junctions exert an impact on suicide-gene-armed virotherapy.^40^ Due to different forms of tumor cell interactions, multiple cell layers, and drug distribution, the bystander effect can differ between 2D and 3D cell cultures.^41^ Indeed, the spread of 5-FU by diffusion into neighboring uninfected tumor cells is more accurately characterized in 3D culture. Furthermore, cells cultured in 3D conditions appear to be less susceptible to drugs.^42^^,^^43^ Thus, the demonstration of a treatment response in 3D cultures can be more relevant and predictive of drug effectiveness. Our results highlight an efficacy of TG6002 combined with 5-FC in spheroids enhancing the demonstration of their oncolytic potency.

Cell lines and xenograft models constitute conventional models to establish efficacy of treatment in oncology. Because of differential gene expression and function in immortalized cells, data obtained do not often match those observed in *in vivo* studies.^44^^,^^45^ Even if mouse xenograft models are more relevant for pharmacodynamic studies than *in vitro* systems, several limitations are well described.^46^ Indeed, about 11% of anti-neoplastic therapies that demonstrate efficacy in murine models have been approved for human use.^47^ In order to improve translational relevance, oncolytic potency was also investigated in canine biopsies from naturally occurring cancers.

The *ex vivo* tumor biopsy culture is a very useful experimental tool that provides an alternative approach to evaluate oncolytic potency of drugs. Tumor biopsies maintained *ex vivo* retain the heterogeneous cell composition, the stromal architecture, and the extracellular matrix of a solid tumor.^48^ Tissue explants harbor immune cells; in organotypic tissue, these cells have been shown to be able to generate an inflammatory environment and even an antiviral response.^49^ Many experimental systems and culture media that preserve neoplastic features have been developed for the long-term maintenance of tumor explants.^50^ Depending on tissue and techniques, the viability of biopsy explants is reported to be several days.

Transduction of VACV was daily assessed by fluorescence microscopy. The increase of fluorescence could reflect the tumor transduction of VACV. This has been confirmed by immunohistochemistry. Little information is available on fresh canine tumor biopsies. Autio et al.^51^ assessed the transduction of an oncolytic Western Reserve VACV in a mammary carcinoma, an osteosarcoma, and a grade II mast cell tumor. Assessment of tumor transduction has been more frequently evaluated in human organotypic slices or tissue explants.^52^, ^53^, ^54^, ^55^

Most primary tumors are difficult to maintain *ex vivo*, which is why cell lines are typically preferred in laboratory research. As tumor lysis is expected with the treatment, pre-analytical requirements were needed. Necrosis of samples can be related to the site of sampling, the culture conditions, the oncolytic potency of TG6002, and the production of cytotoxic 5-FU. To avoid necrosis bias induced by sampling, peripheral parts of the tumor were selected. To evaluate necrosis induced by culture conditions and, thus, provide a better evaluation of the lytic potency of TG6002, uninfected samples were cultured in the same conditions. The percentage of necrosis was about 75% to 100% for explants treated with TG6002 and lower for uninfected biopsies.

This model suffers from several drawbacks.^56^ First, explants do not grow and proliferate like monolayer cell cultures; therefore, they cannot be expanded. Second, even if additional growth factors are added in culture medium, the culture conditions and the absence of efficient vascularization induce spontaneous cell death in the explants. Third, explants are not able to mount an adaptive immune response. The antitumoral activity of the adaptive immune response cannot be evaluated in this model.

In conclusion, this study demonstrates that TG6002 is able to infect and replicate in canine tumor cell lines and is oncolytic in both cell lines, xenograft models, and canine mammary adenocarcinoma samples. These results suggest that an oncolytic VACV expressing the *FCU1* gene may offer an effective treatment in canine mammary tumors. As a first step, evaluations of safety and viral shedding will be performed on healthy dogs before the use of TG6002 in pet dogs. Then TG6002 will be evaluated in dogs with canine breast cancer or other epithelial tumors such as digestive carcinoma, urothelial carcinoma, or head and neck carcinoma. As canine mammary adenocarcinomas are a valuable model for human breast cancer, TG6002 could be a promising therapeutic alternative.

## Materials and Methods

### Cell Culture

Human cervical cancer cell line HeLa, human mammary breast adenocarcinoma cell line MDA-MB-231, canine fibroblast cancer cell line A72, and Vero (African green monkey kidney) cells were obtained from the American Type Culture Collection (ATCC; Rockville, MD, USA). Canine mammary carcinoma cell line REM134 was obtained from the European Collection of Authenticated Cell Cultures (ECACC: Salisbury, UK). HeLa and A72 cell lines were grown in Iscove’s Modified Dulbecco’s medium supplemented with 20% fetal bovine serum (FBS).^21^^,^^22^ REM 134 and MDA-MB-231 were cultivated in Eagle’s minimal essential medium supplemented with 10% FBS. Vero cells, used for virus titration, were cultivated in Dulbecco’s modified Eagle’s medium supplemented with 10% FBS. Primary chicken embryonic fibroblasts (CEFs) were used for recombination, amplification, and production of viral vectors. CEF cells were prepared as previously described and maintained in Eagle-based medium supplemented with 5% FBS.^57^

### Generation of Recombinant VACVs

All oncolytic VACVs were derived from the Copenhagen strain and are deleted in thymidine kinase (*J2R*) and in the large subunit of RR (*I4L*) genes.

TG6002 expressing the fusion gene *FCU1* (Δ*I4L*Δ*J2R*/FCU1 VACV) under the control of the p11K7.5 promoter was constructed and characterized previously.^10^ The same methods were used to generate the double-deleted VACV expressing GFP (Δ*I4L*Δ*J2R*/GFP VACV), designated VVTG17990, by homologous recombination between the previously described Δ*J2R*/GFP VACV and the pΔΙ4L shuttle plasmid containing the selection cassette encoding the guanine phosphoribosyltransferase surrounded by the flanking sequences of VACV *I4L* gene.^36^ TG6002 and VVTG17990 were amplified in CEF and purified, and virus stocks were titrated on CEFs by plaque assay.

### Infection Efficiency on Adherent Cell Cultures

To determine the *in vitro* infection efficiency of double-deleted VACV, 3 × 10^5^ REM134 cells were plated in six-well culture plates and infected with VVTG17990 at MOIs of 10^−3^, 10^−2^, 10^−1^, and 1. Quantitative evaluation of fluorescence was performed 24 h after infection by flow cytometry (BD FACSCanto IITM, BD Biosciences, San Jose, CA, USA). Prior to flow cytometry, single-cell suspensions were fixed with 4% paraformaldehyde (PFA).

### Virus Titration

#### Adherent Cell Cultures

HeLa, MDA-MB-231, A72, and REM134 cells were seeded at 3 × 10^5^ cells per well in six-well plates and infected with VVTG17990 at a MOI of 10^−5^ or 10^−4^. Four days after infection, cells and culture medium were collected and submitted to a quick freeze-thaw cycle and sonication to release intra-cellular viral particles. For viral replication kinetics, 3 × 10^5^ REM134 cells were plated in six-well culture plates and infected with TG6002 at 300 PFUs per well (MOI of 10^−3^). At 1, 2, and 3 days post-infection, the virus titers were determined by plaque assay on Vero cells.

#### Spheroid Cultures

REM134 cells were seeded at 500 cells per well in 96-well ultra-low-attachment plates (Corning, Corning, NY, USA) and allowed to form spheroids for 96 h. Spheroids were then infected with TG6002 at 100 PFUs per well. At 1, 2, 3, and 4 days post-infection, spheroids and supernatant were harvested together and submitted to a quick freeze-thaw cycle and sonication. Viral titers were determined on Vero cells by plaque assay.

### Cell Viability of Infected/Prodrug-Converting Cells

REM134 cells were seeded as described earlier for both adherent and spheroid cultures. Adherent cells were infected with TG6002 at MOIs of 10^−5^, 10^−4^, 10^−3^, and 10^−2^. Five days post-infection, cell viability was determined by trypan blue exclusion, using a Vi-CELL Cell Counter (Beckman Coulter, Brea, CA, USA). For sensitivity to 5-FC, adherent REM134 cells were infected with TG6002 at a MOI of 10^−4^. Two days after infection, the cells were exposed to various concentrations of 5-FC for 3 days before determination of cell viability by trypan blue exclusion. To assay cell viability in the 3D model, REM134 spheroids were infected at 10, 10^2^, 10^3^, and 10^4^ PFUs per well. Two days after infection, 5-FC (at a final concentration of 1 mM) was added, and cell viability was determined 8 days later by a fluorescent assay (CellTiter-Blue, Promega, Madison, WI, USA) using an automated microplate.

### Mouse Experiments

All animal protocols were carried out according to standard operating procedures of the Federation of European Laboratory Animal Science Associations and have been approved by the French Research and Education Ministry. Swiss nude mice were obtained from Charles River Laboratories (Saint-Germain-Nuelles, France). Animals used in the studies were uniform in age (6 weeks) and body weight (20–23 g).

#### Subcutaneous Tumor Model

For immunohistochemical analysis and evaluation of the therapeutic activity of VACVs in the canine xenograft model, 5 × 10^6^ REM134 cells were injected subcutaneously into the flank of the mice. When tumors reached a volume of 100–150 mm^3^, the mice were randomized in a blinded manner and treated with the indicated vectors.

#### Immunohistochemical Analyses of Xenograft Tumors

Immunohistochemical analyses were performed on REM134 tumor xenografts resected from mice injected once with VVTG17990 intratumorally at 10^6^ PFUs or intravenously at 10^7^ PFUs. Tumors were harvested 2 weeks after virus injection and fixed in 4% PFA for 1 h and overnight in 30% sucrose and OCT (coating medium for frozen tissue sections) embedded. To evaluate VACV infection, vascularization, and apoptosis of the tumor, samples were sectioned and stained using the following primary antibodies: rat anti-CD31 monoclonal antibody MC13.3 (dilution 1/500) (557355, BD Biosciences, San Jose, CA, USA), rabbit anti-VACV polyclonal antibody (dilution 1/1,000) (B65101R, Meridian Life Science, Memphis, TN, USA), and rabbit anti-cleaved caspase-3 polyclonal antibody (dilution 1/200) (9661, Cell Signaling Technology, Leiden, the Netherlands). In addition to the immunohistochemistry staining, slides were counterstained with DAPI to label nuclear DNA, and direct detection of GFP expression under fluorescence microscopy on tissue section was done.

#### *In Vivo* Antitumoral Activity of TG6002 in the REM134 Subcutaneous Model

Mice were treated three times intratumorally or intravenously (days 0, 2, and 4) with TG6002 at a dose of 10^6^ PFUs or 10^7^ PFUs, respectively. Seven days after viral infection, 5-FC was given by oral gavage at 100 mg/kg twice daily for 3 weeks. Tumors were measured, and mice were weighed twice weekly for 25 days.

### Canine Mammary Tumor Explants

The study was approved by the Ethics Committee of the National Veterinary School of Alfort (2017-12-01), and all owners had signed the informed consent form. Samples of canine mammary adenocarcinomas were collected immediately after therapeutic surgical excision for three dogs. Canine mammary adenocarcinomas were characterized by Ki67 evaluation. Ki67 antibodies were used on resection specimens. Immunohistochemistry was performed using a DXT automat (Ventana Medical Systems, Roche Diagnostics, Grenzacherstrasse, Switzerland) with the streptavidin-biotin-peroxidase complex method with 3,3′-diaminobenzidine (DAB) as a substrate and hematoxylin counterstaining. The primary antibody was a rabbit monoclonal antibody (Ki67 [SP6], RM-9106-S1, Thermo Fisher Scientific, Waltham, MA, USA) at 1/200. For the assessment of Ki67, the slides were digitized and areas devoid of necrosis were selected. Counting was performed manually on at least 500 tumoral epithelial cells per tumor. The number of tumoral epithelial cells with positive nuclear labeling and the total number of tumoral epithelial cells were calculated via ZEN software (Carl Zeiss Microscopy, Oberkochen, Germany). For each tumor, the Ki67 index was calculated by dividing the number of labeled tumoral cells by the total number of tumoral epithelial cells and expressed as a percentage.

Fresh biopsy samples of canine mammary tumors were collected immediately after surgical excision and incubated in 48-well plates in Iscove’s modified Dulbecco’s medium, 20% fetal bovine serum, 2 mM glutamine, 100 U/mL penicillin, 100 μg/mL streptomycin, and 40 μg/mL gentamycin. Biopsies were incubated at 37°C with a 5% CO_2_ level. Biopsy samples were cut into fragments approximately 8 mm in diameter and 4 mm in thickness. To assess susceptibility of canine mammary tumor explants to VACV, three biopsies were infected with 10^6^ PFUs of VVTG17990 for 4 days. Evaluation of fluorescence was performed every day using a fluorescence microscope (Zeiss LSM 700 confocal microscope, Oberkochen, Germany). An uninfected biopsy was used as control. After 4 days, biopsies were fixed in 10% neutral buffered formalin for 24 h, and immunohistochemical analyses were performed using a rabbit anti-VACV polyclonal antibody (dilution 1/500) (B65101R, Meridian Life Science, Memphis, TN, USA). Immunohistochemistry analyses were performed using a DXT automat (Ventana Medical Systems, Roche Diagnostics, Grenzacherstrasse, Switzerland) with the streptavidin-biotin-peroxidase complex method with DAB as a substrate and hematoxylin counterstaining formalin-fixed tissue sections. Paraffin-embedded HeLa cell lines (3 × 10^5^ cells) infected for 24 h with VVTG17990 at a MOI of 10^−1^ were used as positive control. Evaluation of oncolytic potency of TG6002 was performed by infection of canine mammary tumor biopsies at the dose of 10^6^ or 10^7^ PFUs for 6 days. On the second day of infection, 5-FC was added in the culture medium at the concentration of 10^−2^ M. Six days after infection, biopsies were fixed in 10% neutral buffered formalin for 24 h. Histological analyses were performed on hematoxylin-eosin-saffron-stained tissue sections. Oncolytic potency of TG6002 was determined by the evaluation of tubular necrosis. Slides were evaluated blindly by a board-certified veterinary pathologist. Tubular necrosis was classified into four groups: 0% to 25%, 25% to 50%, 50% to 75% and 75% to 100%.

To assess viability of biopsies in our culture conditions, histological analysis of the control, cultured 6 days in the same condition without TG6002, was performed. To confirm susceptibility of canine mammary tumors to 5-FU, tubular necrosis was evaluated by histological analysis on a sample cultured with 5-FU (10^−2^ M) for 4 days and fixed in 10% neutral buffered formalin for 24 h.

Evaluation of cytosine deaminase activity was also quantified by measuring the amount of 5-FU released in the culture media. After 48 h of infection by TG6002 at 10^6^ and 10^7^ PFUs, 1 mM 5-FC was added to the culture medium containing the biopsies. Every day for 5 days, 5-FC and 5-FU concentrations in the media were measured by high-performance liquid chromatography. Fifty microliters of media were quenched with 50 μL acetonitrile. The samples were vortexed and centrifuged. The organic supernatant was evaporated to dryness and reconstituted in 50 μL water and analyzed by high-performance liquid chromatography using a mobile phase of 50 mM phosphoric acid adjusted to a pH of 2.1. Results are expressed as the percentage of 5-FU relative to the total amount of 5-FC plus 5-FU after various incubation times with 5-FC.

### Statistical Analyses

Statistical analyses of surviving cells were performed using a Student’s t test. A p value <0.05 was considered to be statistically significant. Statistical analyses of tumor volume were performed using the nonparametric Mann-Whitney U test (Statistica v.7.1 software, StatSoft). A p value <0.05 was considered to be statistically significant.

## Author Contributions

J.B., J.F., C.M., E.Q., B.K., and P.E. designed the study and analyzed the data. J.B., J.F., E.L., J.H., V.N., C.P., S.C., P.C., and H.H. performed the experiments. J.B., J.F., C.M., B.K., and P.E. wrote the manuscript.

## Conflicts of Interest

J.B., J.F., J.H., V.N., C.P., S.C., P.C., E.Q., and P.E. were employees of Transgene when the work was performed. Transgene is a publicly traded French biopharmaceutical company, with Institut Merieux as the major shareholder. The other authors declare no other competing interests.
